# Fringe Texture Driven Droplet Measurement End-to-End Network Based on Physics Aberrations Restoration of Coherence Scanning Interferometry

**DOI:** 10.3390/mi16010042

**Published:** 2024-12-30

**Authors:** Zhou Zhang, Jiankui Chen, Hua Yang, Zhouping Yin

**Affiliations:** State Key Laboratory of Intelligent Manufacturing Equipment and Technology, School of Mechanical Science and Engineering, Huazhong University of Science and Technology, Wuhan 430074, China; zhou_zhang@hust.edu.cn (Z.Z.); huayang@hust.edu.cn (H.Y.); yinzhp@hust.edu.cn (Z.Y.)

**Keywords:** droplet measurement, coherent scanning interference, physics aberration, deep learning, inkjet printing, image restoration

## Abstract

Accurate and efficient measurement of deposited droplets’ volume is vital to achieve zero-defect manufacturing in inkjet printed organic light-emitting diode (OLED), but it remains a challenge due to droplets’ featurelessness. In our work, coherence scanning interferometry (CSI) is utilized to measure the volume. However, the CSI redundant sampling and image degradation led by the sample’s transparency decrease the efficiency and accuracy. Based on the prior degradation and strong representation for context, a novel method, volume measurement via fringe distribution module (VMFD), is proposed to directly measure the volume by single interferogram without redundant sampling. Firstly, the 3D point spread function (PSF) for CSI imaging is modeling to relate the degradation and image. Secondly, the Zernike to PSF (ZTP) module is proposed to efficiently compute the aberrations to PSF. Then, a physics aberration restoration network (PARN) is designed to remove the degradation via the channel Transformer and U-net architecture. The long term context is learned by PARN and beneficial to restoration. The restored fringes are used to measure the droplet’s volume by constrained regression network (CRN) module. Finally, the performances on public datasets and the volume measurement experiments show the promising deblurring, measurement precision and efficiency.

## 1. Introduction

**Figure 1:**
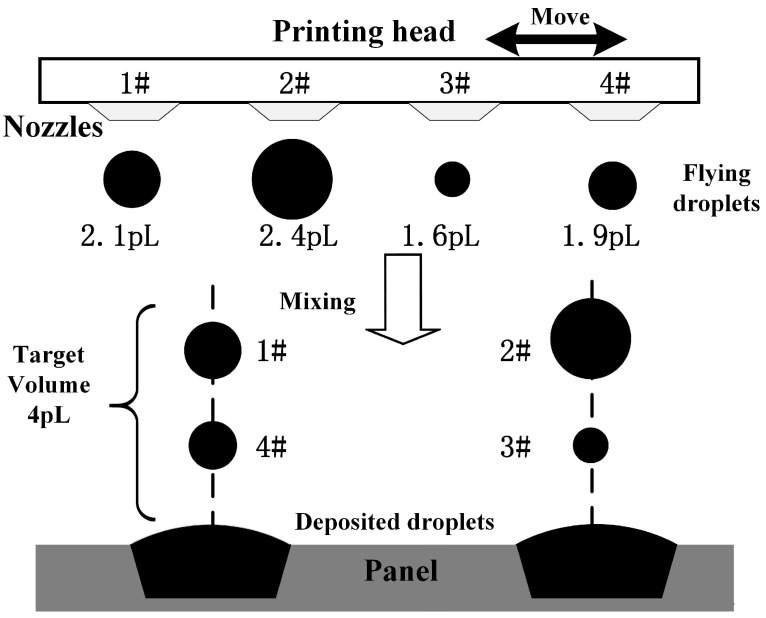
Schematic of inkjet printing in OLED manufacturing. Different jetting volume printing nozzles are planned to spray into the corresponding position to mix up the deposited droplet with target volume by motion planning.

OLED has attracted the focus in intelligent display due to its low consumption of energy, fast response of time and flexibility [1]. Inkjet printing [2], an additive manufacturing for microfluidics fabrication [3], is viewed as a promising fabrication technology for OLED manufacturing [4], hierarchical emulsions fabrication [5] and other field [6] such as solar cells and conductive structures. As shown in Figure 1, there is a chief flowchart of inkjet printing process in OLED manufacturing. Multiply printing heads equipped with thousands of nozzles are employed to boost the production. However, the jetting volume difference between different nozzle can not be completely avoided [7]. For simplification, 4 nozzles 1#, 2#, 3# and 4# are remained shown in Figure 1, whose jetting volume is 2.1 pL, 2.4 pL, 1.6 pL and 1.9 pL separately. The is one hypothetical example to explain how the deposited droplet is generated. The jetting volume 2.1 pL, 2.4 pL, 1.6 pL, 1.9 pL and the target volume 4 pL are the artificial assumption value. In order to achieve the target volume 4 pL in all pixels on panel, the flying droplets jetting from nozzle 1#, 4# is mixed according to the motion planning [8], while it from nozzle 2#, 3# is mixed. In reality, the situation will be more complicated. If the jetting volume can’t be mixed up by the rest nozzles to reach the target volume, the corresponding nozzle will be abandoned. Therefore, the different nozzles’ jetting volume directly affect the motion planning to mix up and the actual volume of deposited droplet. The defects, Mura [9], will exist as the volume of deposited droplets exceed a threshold compared to the target value. Meanwhile, for the sake of high production, the measurement for deposited droplet tends to be real time. Therefore, it’s vital to measure the deposited droplets accurately and efficiently.

**Figure 2:**
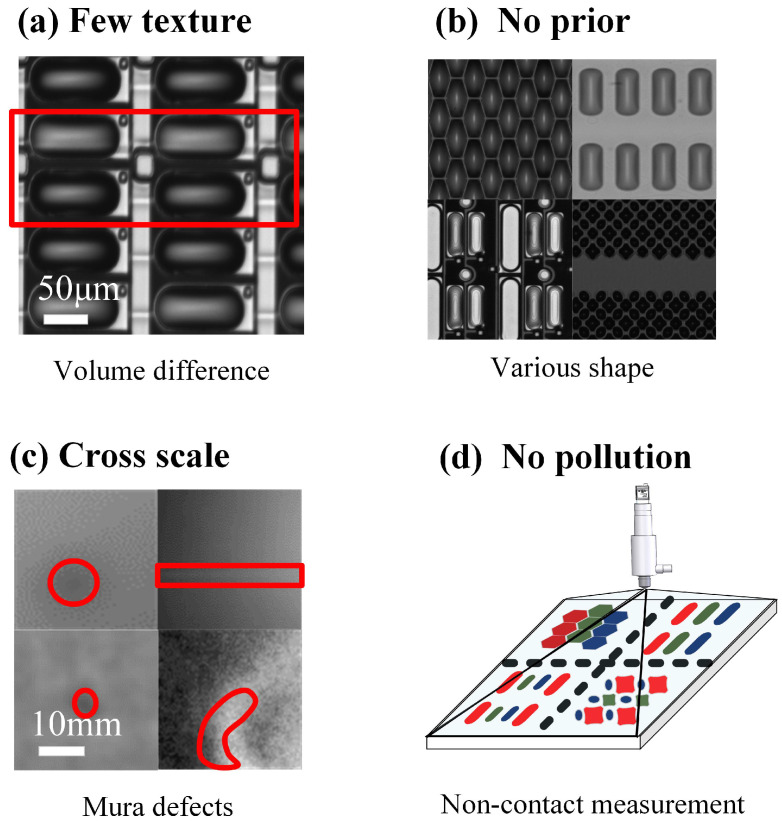
Features of deposited droplets in OLED. (**a**) smooth surface with few texture features. (**b**) droplets with various deposited shapes decided by the shapes of pixels. (**c**) cross scale between the deposited droplets and the panel. (**d**) non-contact measurement for no ink pollution.

To reach the volume uniformity of deposited droplets in inkjet printing, a high measurement error of ±5% for droplet volume is crucial in OLED manufacturing, while every measurement tends to be real time. In addition to the above demands, there exist several challenges in deposited droplet’s volume measurement. There are few texture features to be utilized, and the measurement has no extra information as shown in Figure 2a. The shapes of deposited droplets depend on that of pixels as shown in Figure 2b. Therefore, there are no prior simplified assumptions for the shape of deposited droplets. The common hemisphere assumption is unsuitable. The panel and Mura size are much larger than the droplet size as given in Figure 2c, where exists a conflict between the accuracy and efficiency. In order to measure the volume of deposited droplet in the any place of panel, a global measuring technique is preferable. As shown in Figure 2d, the ink used in OLED is special materials, any pollution leads to the luminescence effect decline and vision measurement methods are needed. Therefore, the measurement for deposited droplets in OLED manufacturing is still a challenge.

Vision based methods are typical non-contact measurement techniques, which can be further classified into: direct and indirect methods. Common direct vision methods mean that the measurement is finished by raw images and is a passive way without extra information. The methods based on stereo vision are wildly used to measurements. Tsai et al. [10] utilizes the depth map to achieve obstacle recognize, which is based on features capturing. Wang et al. [11] empolys the image features to localization and mapping by the stereo vision. Wang et al. [12] establishes a segmented parametric model to measure the radius of multi-bend tubes based on the struct of the bend with stereo vision. All the above methods based on texture or struct features will have a bad performance for deposited droplet’s measurement due to the smooth and featureless surface. In addition, due to the various shapes of deposited droplets, the direct methods have no access to measure the volume by prior shape assumption and direct 2D scale measurement [13]. There are some existing droplet measurement methods based on shape prior information. Liu et al. [7] measures the droplet’s 2D profile in flying situation with 3% accuracy. The laser phase Doppler analysis [14] (PDA) method is efficient, but PDA only measures the droplet size and is based on the sphere assumption to calculate the volume. The measurement error is 6%. The methods based on the ultrasonic attenuation [15] acquire droplet size to acquire 15% accuracy. However, in our condition, the shape of deposited droplet is related to the shape of the pixel. Therefore, indirect methods with extra information are considered. Typically, light [16] or fringe projection profilometry [17] extract the 3D information from the distinction between the input known and output modulated patterns. Although those indirect methods are wildly used, the sizes of the known patterns exceed that of deposited droplet, and the output patterns modulated by droplet will not carry correct height information of droplets. In addition, the common 3D measurement methods are considered, such as coherent scanning interferometry (CSI), focus variation microscopy (FVM) and confocal microscopy (CM). All of them have high accuracy and been wildly used in the field of medicine, biology and industry to measure the roughness or complex micro-structure. Every method has its limit and advantages [18]: CM measures greater height than the others, while CSI measures greater heights than FVM. In the repeatability of heights measurement, CSI has the largest bias while FVM achieved the lowest bias. As for disturbance, CSI has the more sensitivity to variations than the others. As for the measurement noise between different methods, Both CSI and FVM have the smallest and similar measurement noise, while CM has higher noise value. In detail, the FVM [19] utilizes the limited depth of focus to scan the sample, and acquire the metrology from the variation of focus. Based on the maximum measurable slope angle and true color for every measurement point, FVM is wildly used to measure the steep surface flanks or the roughness. However, the transparent specimens, such as the deposited droplet smooth surface, will cause complex rebuilding problems for FVM. The CM [20] only allows the desired light to pass through via a small pinhole, while the light beyond the focal plane is abandoned. The focal spot scans the whole x-y plane to cover all points on image plane. With the aid of vertical scanning, the CM can cross the range of the surface. The imaging acquired speed is limited. When measuring transparent material, there may occur the interference changing the intensity recorded and lead to the error positioning in height. As the information in the recorded image is only related to the exactly focal point lied at surface, the image is less informative in the dimension of x-y plane to analyze the interference. CSI utilizes the interference to locate the surface via changing the optical path difference between the sample and optical system, which can measure roughness, discontinuous steps and transparent samples with nano resolution [21]. Even there are physics aberrations when the transparent samples have no parallel surface, the post process implementation for interferograms to eliminate physics aberrations has more possibility. Besides, CSI has the advantages: the global measurement, without shape assumptions for the sample, non-contact measurement. Therefore, in this paper, CSI is chosen as the measurement techniques, which is great potential for deposited droplet volume measurement.

CSI has been widely used in 3D topography. However, the physics aberrations lead to the image degradation and degrade the effect of CSI. The image depends on the effect of CSI optical focus and phase delay led by the sample, and both will introduce aberrations and cause the degradation in image. Several methods have been proposed to detect the aberrations. The ideal imaging is influenced by the aberrations and the image quality is decided by PSF. The key to restore the aberrations is to extract the correct PSF. Gaussian model [22], Gaussian mixture model [23] and dual-pixel model [24] have been utilized to describe the defocus aberration. Although they have achieved great performance, they only focus on defocus aberration and is inappropriate for the real-world aberration. Contrary to the modeling methods, experiments based methods [25,26] are proposed and likely the calibration for the physics aberration. The study [27] measured the PSF via capturing the light from the ideal point light. In addition, deep learning methods [28,29,30] are employed to detect the physics aberrations. As the aberrations led by the samples are dynamic, those above methods are not adaptive and have trouble in describing the sample-induced aberrations. In this paper, a dataset of random aberration composed of up to 34 Zernike polynomials is established. The VMFD is trained to extract the estimated PSF via deconvolution to restoration.

Image blurring can be regarded as the convolution of PSF and sample, where PSF reflects the response of optical imaging system to single point imaging. In order to achieve the aberrations elimination, the existing researches are widely based on the deconvolution idea, which converts the blurring image after PSF convolution into a clear image by deconvolution, so as to restore the imaging effect. As the deconvolution process is a non-convex optimization problem, there needs extra constraint guidance. The dark channel prior [31] is proposed to deblur the image. Combined with the spatial prior and the iterative support detection, an effective kernel estimation method [32] was proposed for motion deblurring and non-blind deconvolution. Wiener filter and TSVD decomposition [33] was discussed in image restoration process. A multi-stage strategy [34] for image restoration was proposed based on the optimal balance between spatial details and the context information. On certain scenarios, the above approaches will achieve a good results. Based on the image prior or constraint, the existed methods [35,36,37] reached the valid solution space. However, a proper prior is hard to design and commonly not generalizable. To address those problems, in recent years, the methods based on deep learning, with the estimation of PSF or fuzzy kernel, have achieved great success in image restoration. Based on the convolutional neural networks (CNN) [38,39,40,41], the general kernels have been learnt. CNN-based methods achieved strong performance via novel design, such as dilated convolutions [42], attention modules [43] and the encoder-decoder structure [44]. Besides, the wildly used U-net architectures [24,45,46] have shown the strength in features extraction in the task of image restoration. Besides, Transformer [47,48,49] has achieved great success in image restoration. However, the multi-heads designs of Transformer leads to be time consuming [50].However, those deep learning methods’ performances depend on the prior designed. There is less interaction between the unknow sample-induced aberrations and restoration ability. On contrary to the existing methods, VMFD based on channel Transformer and U-net architecture creates the interaction between aberrations and restoration to make full use of the priors.

By the analysis for fringe texture, CSI rebuilds the 3D topography of sample with accuracy [51], where the change of fringe pattern is related to the height of sample. In order to acquire the whole change of fringe, CSI scans the sample in the height direction. On one hand, the reference beam remains the same on phase and amplitude. On another hand, the phase of object beam reflected from the sample varies with the scanning process, where the phase represents the height information of sample at different scanning position. As the reference beam and object beam are coherent, the interference occurs and the intensity of interferogram depends on the phase difference. According to the sampling theorem, the CSI has to record hundreds of fringe images (interferogram) during the scanning process. Zhang [52] measured the volume of droplet accurately by CSI based on hundreds of fringe images. The scanning distance covers the height of sample, and the time consumption of the whole scanning process is about 5 s. The recording process of interferograms is time-consuming. Besides, the droplets are transparent. The object beam reflected from the sample carries the different surface of droplets leading to the signal overlap and chromatic dispersion. Despite that the CSI has the potential to measure the volume of deposited droplets, there are several problems needed to be handled.

**Figure 3:**
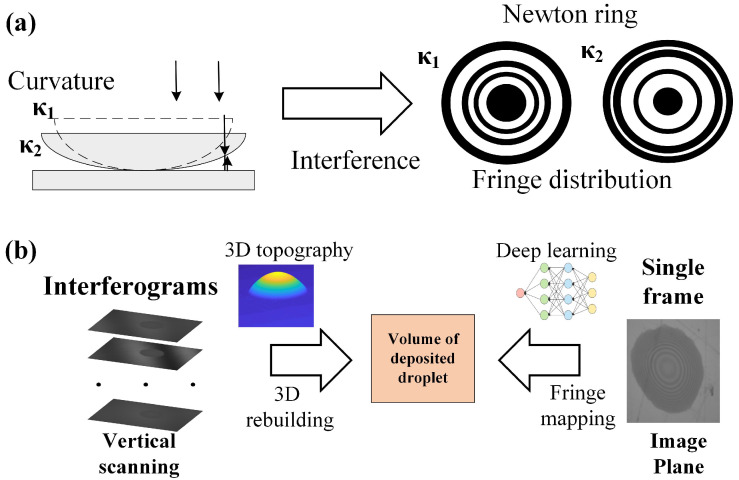
(**a**) Measuring the curvature by the fringe distribution of the newton ring; (**b**) Measuring the volume of deposited droplet by 3D rebuilding based on redundant sampling or fringe mapping based on one single interferogram.

In this paper, volume measurement via fringe distribution network (VMFD) is proposed. Kathryn J [53] utilized the newton ring analysis to measure the thickness of film. Compared to the non-contact interference methods, the method based on newton ring analysis efficiently acquired the consistent results. As shown in Figure 3, according to the newton ring, the curvature of different surface can be determined. Learning from the newton ring analysis, VMFD builds the mapping relation between the fringe distribution and the final volume. As known, the fringes in interferograms reflect the relative height of the corresponding point and show the curvature variation of droplet’s surface. At the same time, the droplets’ deposited shape is decided. Therefore, the fringe distribution from the droplets’ surface is positively related to the droplets’ volume, where single interferogram with fringes is enough to measure the volume by VMFD. Without the recording process in tradition way, the measurement will be efficient.

**Figure 4:**
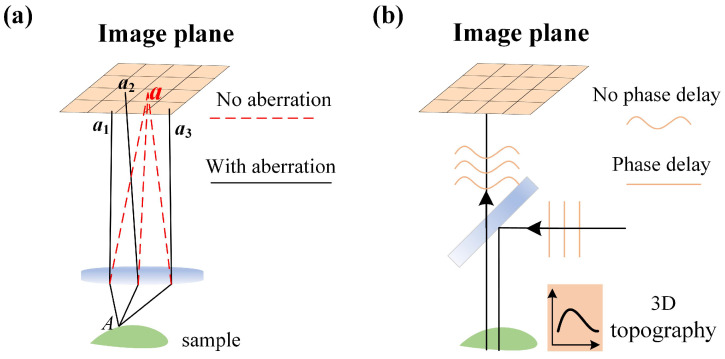
The physics aberrations led by (**a**) the optical system and (**b**) the characteristics of the transparent sample.

Different from the natural newton ring analysis to measure the curve, with the aid of CSI, the droplet’s surface is full of texture features and the fringes from the smooth and continuous surface in interferogram occur artificially. Besides, there are aberrations caused by optical system and the sample. As shown in Figure 4a, due to the dispersion led by the lens in CSI system, the image recorded will be blurred, where the light reflected from the point A in sample will accumulate at different points on the image plane. Owing to the transparency of droplet, the phase of reference light will be delayed and the image is with aberration as shown in Figure 4b. Zhang [54] analysed the distortion led by the transparent sample in non-contact interference measurement and proposed the diffusion model to iteratively remove the distortion, but the process was carried on the scanning direction and still time consuming, which is not satisfactory in efficient measurement. Therefore, the aberrations can not be avoided during the analysis for the fringes in interferogram, and the aberration restoration remains complex and challenging.

Based on the above comments, VMFD takes a fringe image as the input to avoid the recorded process to be real time. Firstly, the 3D point spread function (PSF) is built to model the link between the image degradation and physics aberration. Secondly, in order to present the physics aberration, the Zernike polynomial is utilized and the Zernike to PSF (ZTP) net is proposed to efficiently transfer the physics aberration to PSF. Then, a physics aberration restoration network (PARN) is designed to remove the degradation. Combined with the designed channel Transformer module and U-net architecture, PARN can perform well. After the aberration led by the transparency of deposited droplet is restored, according to the newton ring analysis, the fringe texture features of interferograms are employed to measure the volume of deposited droplet by designed constrained regression network (CRN).

Overall, the main contributions are listed as follows.

- An efficient volume measurement method, VMFD, is proposed based on the non-contact measurement CSI. Learned from the newton ring analysis, VMFD measures the volume via just one single interferogram end to end. The interferogram acquired by the CSI is full of fringe texture features. Without hundreds of interferograms needed in tradition way, the whole measurement becomes efficient.
- In order to better describe the aberration according to the physics information in the real situation, the Zernike polynomial is employed. Unlike the common vision system, CSI is a 3D measurement technique. A 3D PSF for CSI is built to link the relations between the aberration and image degradation.
- Technically, a simple but effective method, ZTP, is proposed to efficiently transfer the coefficients from the Zernike space basis to PSF space basis, and the complex calculations by 3D PSF are avoided.
- Based on the prior degradation information and strong representation for context by Transformer module, PARN is proposed and has the promising effect in aberration image restoration.
- A encoder-decoder structure, CRN, is proposed and learns the constant features from the fringe distribution with the weight of other factors decline.

The remainder of this article is organized as follows. In Section 2, related works are introduced concerning the transparent sample measurement methods based on CSI and multi-gaussian model for signals separation. In Section 3.1, the theoretical analyses of overlapped fringe signals are employed, and WTCSI is proposed in detail, including overlapped fringe signals analyses, envelope detection, envelope distinction, envelope separation, and zero-order fringe peak recognition. In Section 3.2, experiments are designed to verify the performance of the proposed WTCSI method, and the results are discussed. In Section 4, the conclusions are presented.

## 2. Methodology

In this section, the analysis of VMFD is briefly discussed. The fringe texture pattern image for one measurement is recorded by CSI. After the blurring image through VMFD, the volume result of deposited droplet is achieved end to end. This work concentrates on image restoration and volume measurement, and the original value of volume is acquired in our previous work [52,54]. Firstly, the 3D PSF for CSI system is built. Then, the architecture of VMFD is discussed. Next, the backbone networks ZTP, PARN and CRN along with the key modules are presented in detail.

### 2.1. The 3D Imaging Degradation Model for CSI

**Figure 5:**
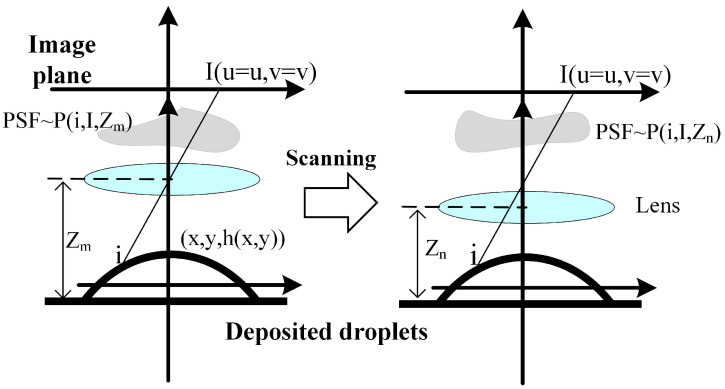
The imaging process for deposition droplet interferogram sequence.

As shown in Figure 5, different from common imaging techniques, CSI scans the deposited droplets and obtains a series of interferograms of the same deposited droplets at different scanning heights, which is essentially a 3D spatial imaging process. Therefore, the axial scanning height is also a key factor in interferogram imaging.

The world coordinates of the deposited droplet morphology can be described as below.

(1) $$S_{3D}\left(x,y,x\right)=A\left(x,y\right)\delta \left(z-h\left(x,y\right)\right)$$

where A(x,y) represents the intensity after the interference. δ() is the impulse function. h(x,y) is the deposited droplet morphology. Any point in morphology can viewed as i(x,y,z=h(x,y)), the light from it projects onto the image plane as I(u,v) through the 3D PSF. The PSF is not only related to the spatial position, but also the scanning height, and regarded as p(i,I,Z). *Z* represents the arbitrary scanning height $Z_{m}$ or $Z_{n}$. The real intensity recorded on the image plane can be discribed as below.

(2) $$S_{2D}\left(I\right)=\;\int \int \int \;S_{3D}\left(x,y,x\right)p\left(i,I,Z\right)dxdydz$$

As the morphology of the deposited droplets is known, Equation (2) is reduced to the below.

(3) $$\begin{array}{cc} S_{2D}\left(I\right) & =\;\int \int \int \;A(x,y)\delta (z-h(x,y))p(x,y,z=h,I,Z)dxdydz\\ \; & =\;\int \int \;A(x,y)p(x,y,I,z)dxdy \end{array}$$

where p(x,y,I,Z) is the 3D PSF. The 3D PSF builds the relations between the coordinate (x,y) and the image plane coordinate I(u,v) to represents the spatial variant characteristics, where the complex kernel can be modeling. More importantly, the blur at different scanning height is considered.

In order to describe more complicated aberrations, the Zernike polynomials are utilized.

(4) $$\psi \left(p,Z\right)=\lambda \left(p,Z\right)\sum_{n,m}a_{nm}Z_{n}^{m}\left(P\right)$$

where $Z_{n}^{m}$ is fundamental polynomial. $a_{nm}$ is the coefficient of Zernike polynomial [55,56]. p is the coordinates of any point in a polar coordinate system with aperture *D*. *Z* is the scanning height. λ(p,Z) is the coefficient to represent the aberration weights. Through the Fourier transform, the aberrations represented by the Zernike polynomials can be viewed as the 3D PSF as below.

(5) $$p\left(x,y,I,Z\right)=|FW(pe^{-i\psi (p,Z)}){|}^{2}$$

where W() is the pupil function. F is the Fourier transform.

### 2.2. The Framework of VMFD

**Figure 6:**
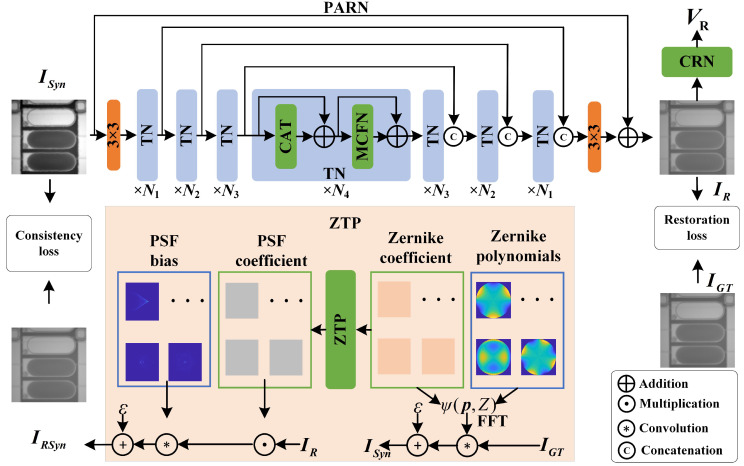
The framework of VMFD. In VMFD, there are three modules ZTP, PARN and CRN. ZTP modules transform the phase aberrations to PSF. PARN is trained to eliminate the deblur. CRN is utilized to measure the volume via single restored interferogram.

As shown in Figure 6, the overall framework is divided into three parts: ZTP, PARN (Channel Attention Transformer + Multi Convolution Forward Network, CAT + MCFN), CRN. In order to describe the aberrations of the CSI system in the measurement of deposited droplets, ZTP is proposed, which maps the aberrations to the PSF of the spatial domain based on the Zernike polynomials, and avoids the complex calculation of Fourier transform to improve the efficiency. The PARN extracts the deep features from the blurring images and realizes the aberrations removal through multi-channel feature fusion and residual analysis, and restores the original fringe distribution. According to the single interferogram full with fringe textures, CRN achieves the end-to-end volume measurement.

After the blurry image $I_{Syn}\in R^{H\times W\times 3}$ is determined, PARN restores the input image based on the features extracted by the aberrations, and outputs the restored image $I_{R}\in R^{H\times W\times 3}$, where *H* and *W* represents the height and width of the image. In order to eliminate aberrations through PARN, ZTP is designed as Equation (6) to generate different aberrations to make images degraded.

(6) $$I_{Syn}=f_{ZTP}\left(I_{GT},\mathrm{Hyp}\right)+\varepsilon$$

where $f_{ZTP}$ is the process of ZTP to degrade the image with arbitrary aberrations. $\varepsilon \in R^{H\times W\times 3}$ is random noise. Hyp is the hyperparameter vetor, including the relative distance between the CSI and the sample, the focal length and the aperture size etc. Accoring to the set up for Hyp, different physics aberrations will be generated.

In the process of PARN, $I_{Syn}$ firstly go through the 3×3 convolution, and the shallow features $A_{0}\in R^{H\times W\times C}$ is acquired to increase the channel dimension. The shallow features pass through the U-net architecture to extract multi-scale deep features. According to the different depth of the network, the image is downsampled with the base of 2, and the channel dimension rises, and the features obtained are represented as $A_{N=l}\in R^{\frac{H}{2^{l-1}}\times \frac{W}{2^{l-1}}\times 2^{l-1}C}$. After that, the deep features are upsampled symmetrically and the channel dimension decrease. l∈[1,4] represents the depth. The above process can described as below.

(7) $$\left\{\begin{array}{cc} A_{N=1} & =f_{TN}\left(A_{N=0}\right)\;l=1\\ A_{N=l} & =f_{TN}\left(A_{N=l-1}\right)\;l=2,3,4 \end{array}\right.$$

where $f_{TN}$ represents the Transformer modules. Each deep feature passes through Transformer module to build channel attention. After multi-scale feature extraction and the same depth features fusion, the process of upsampling is completed on the basis of 2.

(8) $$\left\{\begin{array}{cc} A_{N=l-1}^{\prime } & =f_{TN}\left(A_{N=l}^{\prime }\right)\;l=2,3,4\\ A_{N=0}^{\prime } & =f_{TN}\left(A_{N=1}^{\prime }\right)\;l=1 \end{array}\right.$$

where $A_{N=l-1}^{\prime }\in R^{2^{l-1}H\times 2^{l-1}W\times \frac{C}{2^{l-1}}}$ the features in the upsampling process. After the final convolution and the residual connection with the input $I_{Syn}$, $I_{R}\in R^{H\times W\times 3}$ is acquired. The process of upsampling and downsampling is achieved by the pixel shuffle and pixel unshuffle. The whole process of PARN can be viewed as below.

(9) $$I_{R}=f_{PARN}\left(I_{Syn}\right)+I_{Syn}$$

As the aberrations are eliminated by PARN, CRN is designed to measure the volume of deposited droplet by single interferogram full with fringe texture.

(10) $$V_{R}=f_{CRN}\left(I_{R}\right)$$

where $V_{R}$ represents the value of droplet’s volume.

### 2.3. The Structure of ZTP

In order to improve the computational efficiency of transferring the aberrations to PSF and avoid time-consuming Fourier transform calculation, the key is to establish the mapping between the Zernike polynomial coefficients to PSF coefficients, where the dot product results of each coefficient and each basis are the corresponding aberrations and PSF. Since PSF function is calculated pixel-by-pixel, and PSF needs to be recalculated under different conditions. In order to simplify the complexity, the function basis is established by referring to the PSF basis function method proposed by Mao et al. [57]. The standard Gaussian function is taken as an ideal PSF without aberration and multiplied with the generated space-dependent tilt correlation matrix to obtain the tilt aberration. By multiplying the standard Gaussian function with the higher order aberration correlation matrix [58], the description of the higher order aberration is obtained. According to the setting of different aperture and distance parameters, the PSF database of different shapes is obtained, and the function basis is extracted based on principal component analysis. Therefore, the Equation (5) is transfered to the below.

(11) $$|FW(pe^{-i\lambda \left(p,Z\right)\sum_{n,m}a_{nm}Z_{n}^{m}\left(P\right)}){|}^{2}=\sum_{m=1}^{M}\beta_{m}\omega_{m}$$

where the relations between the Zernike coefficient $a_{mn}$ and PSF coefficient $\beta_{m}$ are established. $\omega_{m}$ is the bais of PSF. A simple but effective neural network is trained to map $a_{mn}$ to $\beta_{m}$. N=36 is enough to represent the most aberrations. M=100 is the numbers of bais in $\omega_{m}$.

### 2.4. Overview of the Proposed PARN

Due to the aberration of the lens in the interference vision system and the non-uniform optical path introduced by the transparent sample, the interference fringe distribution with wavefront loss is captured on the final image plane. Based on the excellent multi-scale analysis characteristics of U-net architecture and the long history modeling capability of Transformer, PARN constructs a restoration network based on the attention mechanism of channel dimensions. As shown in Figure 6, each layer of the four-layer network is configured with a different number of Transformer network (TN) modules. $\left\{N_{i}|i=1,2,3,4\right\}$ is 4, 6, 6, and 8 separately. The number of multihead self-attention in each layer of TN is set to 1, 2, 4, and 8.

**Figure 7:**
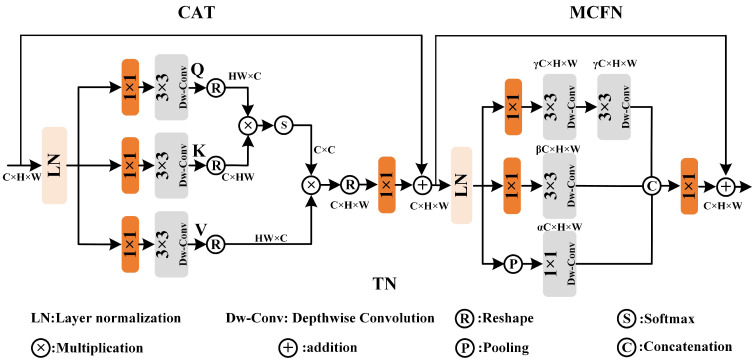
Scheme of TN (CAT+MCFN). PARN is based on the U-net architecture composed of TN blocks to deblur in different scale via channel Transformer.

To avoid redundant computation of attention mechanisms, referring to Zamir et al. [49] channel attention, the computation of Transformer module is reduced to linear complexity, and is restricted to calculate the weight matrix between channels. in Figure 7, TN contains CAT and MCFN modules. The $N_{1}$ layer is taken as example to Illustrate the process of TN. The shape of input is $A_{0}\in R^{H\times W\times C}$. After the layer normalization, the input features go through 3 1×1 convolutions to increase the channel dimensions. After the different branch is operated by different Dw-Conv and reshaping, the corresponding matrixs, Q∈HW×C, K∈C×HW and V∈HW×C in attention mechanism are acquired. The attention Atten is acquired as below.

(12) $$Atten=V\cdot S(\frac{K^{T}Q}{\alpha })$$

where S() is Softmax function. α is the learnt scale factor to prevent the activation function gradient from disappearing. The process of CAT can be regarded as below.

(13) $${\bar{A}}_{N=0}=Conv\left(Atten\right)+A_{N=0}$$

where Conv() is 1×1 convolution. Based on the inception [59] modules’ forward network, multiple scales fine features are extracted. The process of MCFN can be viewed as below.

(14) $${\tilde{A}}_{N=0}=f_{MCFN}\left({\bar{A}}_{N=0}\right)+{\bar{A}}_{N=0}$$

where $f_{MCFN}$ represents the process of multi-scale extraction feedforward network.PARN combines the excellent modeling ability of Transformer with the efficiency of channel dimension calculation and the multi-scale feature extraction characteristics of CNN to eliminate aberrations.

### 2.5. The Structure of CRN

**Figure 8:**
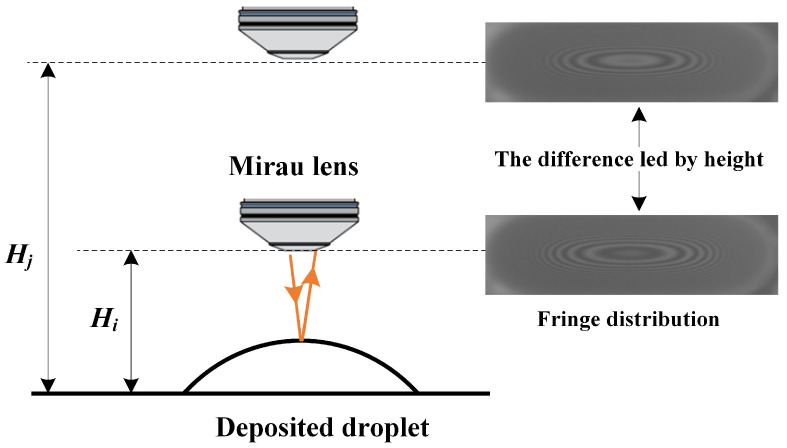
The difference of fringe distribution in single interferogram at different sampling heights.

By analyzing the fringes in interferogram, the curvature variation of the deposited droplets can be determined like the newton ring analysis. Since the deposited region of the droplet is limited to the pixel pit, the volume of the deposited droplet is determined by the topography curvature. Therefore, CRN can achieve one-to-one mapping between the fringe distribution and the volume of droplet. In actual conditions, during the acquisition of interference fringe images of deposited droplets, the relative height between the Mirau interference objective lens and the deposited droplet cannot be guaranteed to be consistent. The optical path of the measured beam reflected from the deposited droplet is not only related to the topography of the deposited droplet, but also to the relative working distance between the lens and sample. Therefore, in order to obtain the volume of deposited droplet from the fringe distribution in a single image, it is necessary to consider the variation of fringe distribution caused by the relative working distance between the Mirau interference objective lens and the sample. As shown in Figure 8, the same deposited droplet is sampled by CSI at height $H_{i}$ and $H_{j}$, and the interferogram with different fringe distribution is obtained separately. In other words, the volume of deposited droplet and the relative height of sampling jointly determine the actual interference fringe distribution. Therefore, in order to obtain the fringe distribution only related to the volume of deposited droplets, the CRN needs to remove the fringe differences caused by different sampling heights.

**Figure 9:**
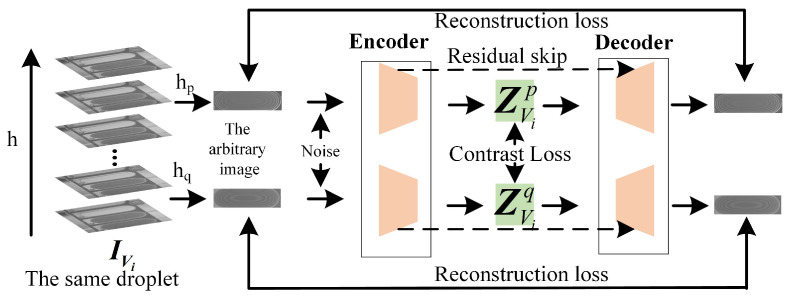
The encoder-decoder structure of CRN.

Based on the above analysis, in order to extract features reflecting fringe differences caused by volume differences, CRN adopts an encoder-decoder structure to achieve strong correlation feature extraction between volume and fringe distribution. As shown in Figure 9, the input of CRN is the fringe dis of the same deposited droplet at different scanning heights. In order to extract features only related to the volume of deposited droplets, the output of encoder-decoder structure needs to be as little different as possible. After the image of the same deposited droplet is sampled at any scanning position, the redundant interferogram sequence, $\left\{I_{V_{i}}|I_{V_{i}}^{1},I_{V_{i}}^{2},I_{V_{i}}^{3}\ldots I_{V_{i}}^{m}\right\}$, is obtained. $V_{i}$ is the volume of droplets corresponding to the redundant interferogram sequence. *m* is the number of image in sequence. According to the above steps, the redundant interferogram sequences acquisition of deposited droplets at different substrate locations and different volumes are completed to be regarded as $\left\{I_{V}|I_{V_{1}},I_{V_{2}},I_{V_{3}}\ldots I_{V_{n}}\right\}$. After randomly choosing any two interferograms $I_{V_{i}}^{p}$ and $I_{V_{i}}^{q}$ from the same $I_{V_{i}}$ along with different degrees Gaussian noise as the input of the same encoder, the corresponding latent space features $Z_{V_{i}}^{p}$ and $Z_{V_{i}}^{q}$ are obtained, where *p* and *q* represent the height $h_{p}$ and $h_{q}$ separately. Then the compressed latent space features are through the same decoder to recover the interferogram without noise. The above training process can be regarded as below.

(15) $$\left\{\begin{array}{cc} Z_{Vi}^{p} & ={\bar{f}}_{CRN\;\_En}\left(I_{Vi}^{p}+\varepsilon_{p}\right)\\ {\tilde{I}}_{Vi}^{p} & =f_{CRN\;\_De}\left(Z_{Vi}^{p}\right) \end{array}\right.$$

where p∈[1,m] and *m* is the number of images in $I_{V_{i}}$. ${\bar{f}}_{CRN\;\_En}$ is the encoder of CRN. $f_{CRN\;\_De}$ is the decoder of CRN. $\varepsilon_{p}$ is the Gaussian noise. By extracting the feature of the interferogram sequence sampled at any different position in the same redundant interferogram sequence, the feature difference caused by the different sampling height is reduced based on the contrast loss computation, and finally the fringe distribution are only related to the droplet volume. Further, in order to obtain meaningful compressed features, decoder needs to complete the image recovery by the collected latent space features, and ensure that the extracted features represent the fringe distribution characteristics in the interferogram through reconstruction loss computation.

**Figure 10:**
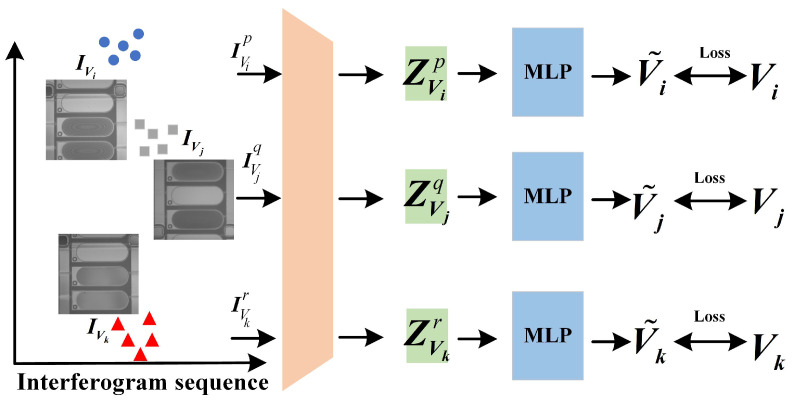
The volume measurement regression network of CRN.

After the above training, the encoder can achieve the clustering of fringe distribution features at different sampling positions in the redundant interferogram sequence. Due to the existence of reconstruction loss, the clustering features still have a certain distance in the latent space. In order to further reduce the volume regression error, on the basis of the above structure, the encoder is retained and the decoder is replaced with the multi-layer perceptron (MLP) structure. As shown in Figure 10, the encoder trained in the previous stage is used to analysis the fringe distribution from single frame interferogram of any volume $I_{V_{i}}$, $I_{V_{j}}$, $I_{V_{k}}$ at any sampling position $I_{V_{i}}^{p}$, $I_{V_{j}}^{q}$, $I_{V_{k}}^{r}$. CRN extracts the corresponding latent space features $Z_{V_{i}}^{p}$, $Z_{V_{j}}^{q}$, $Z_{V_{k}}^{r}$, where *p*, *q*, *r* represent different sampling height. The latent space features are directly input into the MLP to obtain the final calculated volume. Compared the calculated volume and the actual volume value, the final the encoder $f_{CRN_{E}n}$ is obtained. The whole process can be regarded as below.

(16) $$\left\{\begin{array}{cc} Z_{V_{i}}^{p} & =f_{CRN\;\_En}\left(I_{V_{i}}^{p}\right)\\ {\tilde{V}}_{i} & =f_{CRN\;\_MLP}\left(Z_{V_{i}}^{p}\right) \end{array}\right.$$

where $f_{CRN\;\_MLP}$ is the MLP to measure the volume.

After the training of the above two stages, the encoder can effectively extract the fringe distribution features in the interferogram and eliminate the fringe differences caused by the difference of sampling height. In the first stage, the encoder is trained to extract the most critical universal features in the interferogram, and the ability to extract the features with fringe changes caused by volume difference is increased by the contrast loss. In order to avoid overfitting and ineffective training through a single contrast loss, the corresponding decoder is constructed to transfer the latent space features to the interferogram, and ensures that the extracted features are important features of the interferogram. In the second stage, in order to reduce the characteristic difference of interferogram with different sampling heights of the same deposited droplet under the constraint of reconstruction loss, the encoder trained in the first stage is combined with the MLP structure to achieve the direct calculation of deposited droplet volume.

## 3. Experimental Results and Discussion

In this section, the proposed method is inspected via the performance on image restoration tasks and the volume measurement experiments. Images are randomly cropped to image patches. The optimizer employed is Adam, where the $\beta_{1}$ = 0.9 and $\beta_{2}$ = 0.999, The learning rate is gradually updated with the cosine annealing scheduler. All experiments are conducted with NVIDIA RTX 3090 GPUs using PyTorch 1.12 and Python 3.8. The comparisons metrics are using the PSNR and SSIM.

As the aberrations are hard to acquire, we choose the Zernike polynomials to simulate according to the simulation for atmospheric turbulence. With the Zernike basis ${\left\{Z_{j}\left(p\right)\right\}}_{j=1}^{M}$ and coefficients ${\left\{a_{j}\right\}}_{j=1}^{M}$ known, the phase for the aberrations can be expressed as:

(17) $$\begin{array}{c} \phi \left(Rp\right)=\sum_{j=1}^{M}a_{j}Z_{j}\left(p\right) \end{array}$$

where *R* is the apeture radius, p=[p,θ] is the polar coordinate. As the Zernike basis is known, the coefficients are required to represent the aberrations and have geometric interpretations [58]. According to the orthogonality principle, the coefficients can be calculated as:

(18) $$\begin{array}{c} a_{j}=\int_{0}^{2\pi }\int_{0}^{1}W\left(p\right)\phi \left(Rp\right)Z_{j}\left(p\right)\;dp\;d\theta \end{array}$$

where W(p) is the pupil function. Since $Z_{1}\left(p\right)=1$, it states that $\int W\left(p\right)Z_{j}\left(p\right)\;dp=\int W\left(p\right)Z_{j}\left(p\right)Z_{1}\left(p\right)\;dp=0$. Taking the expectation [60] $E[a_{j},a_{j^{\prime }}]$ to describe the correlation between $a_{j}$ and $a_{j^{\prime }}$ and can be calculated by Kolmogorov structure function along with Wiener-Khinchin theorem. After the expectations are acquired, the covariance matrix C can be defined as:

(19) $$\begin{array}{c} C_{j,j^{\prime }}=E\left[a_{j},a_{j^{\prime }}\right] \end{array}$$

In order to draw correlated samples from C, the covariance matrix is decomposed as $C=RR^{T}$. Then the orthogonality coefficients is obtained by:

(20) $$\begin{array}{c} a=Rb \end{array}$$

where b∼N(0,I) s random Gaussian vector. Once coefficients are acquired, the phase representing for the aberrations are obtained. The diversity of aberrations represents the light pass through the refractive media with different optical path difference. The optical path difference is related to the phase. Therefore, we can simulate the aberrations is led by the measurement light beam passing through the deposited droplet.

### 3.1. Performance of Proposed Method on Public Datasets

In order to calculate the volume of deposited droplet via the fringe distribution, PARN firstly removes the image blur caused by the transparent sample and optical system. The deblurring performance decides the accuracy of volume measurement. In this paper, the synthetic image dataset generated from DIV2k [61] are employed as well, while BSD100 [62], Set5 [63], Set14 [64] are utilized as test sets. The generated PSF is decided by ZTP module via Zernike simulator [58]. The optical parameters are corresponding to the real condition. The lens used is Nikon double beam interference objectives. The magnification is 50. The focal length is 4 mm. The wavelength is in the interval from 525 nm to 675 nm. In order to simulate the 3D PSF during scanning process, the real distance is in the interval from 3390μ m to 3410μ m, where the nominal working distance is 3400μ m. As the scanning step is nanometer, the simulator step is set to 10nm to cover more different scanning position.

The comparisons results are listed in Table 1. Due to the random aberrations in degradation, both PSNR and SSIM is relatively not high. In datasets DIV2k, Set5 and Set14, PARN has the best performances. In dataset BSD100, PARN achieves the second best perfermances. Specially, PARN demonstrates the better perfermances with common margins of 0.17 to 0.30 dB. Compared to nonblind method DISCNet, PARN achieves 1.69 dB improvement on dataset DIV2k. Compared to CNN based method SRN, PARN yields 3.00 dB on DIV2k due to the Transformer module’s strong representation ability. Besides, PARN outperforms 0.3 and 0.5 dB than Transformer based methods Uformer and Restormer on DIV2k. Overall, PARN reaches the best perfermances due to the special designed modules, which can better efficiently remove the degradation.

Due to the limited representation ability of convolution, SRN and DISCNet gain relatively low PSNR and SSIM. Based on RNN’s representation for longer context, MTRNN gains more improvements than CNN based methods. With multi-stage context features fusion, MPRNet achieves improvements than RNN based method. The key to deblurring is to completely learn the context. The methods based on Transformer, Uformer and Restormer, are employed to yield better performance, where Transformer are better to capture long-range dependencies owing to self-attention mechanisms. Besides, with the aid of prior information for degradation, DeblurGAN-v2 outperforms than CNN based methods. Therefore, PARN explicitly utilize context features, multi-stage process and attention mechanisms to achieve the best performance.

**Figure 11:**
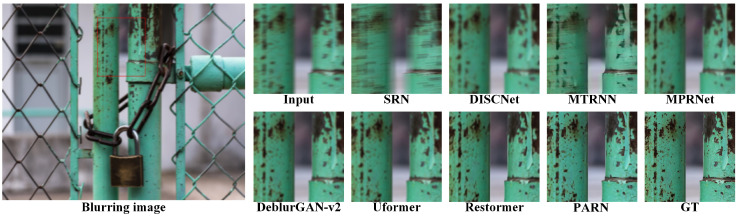
Visual comparisons on the datatset DIV2K [61]. PARN has the better restoration.

**Figure 12:**
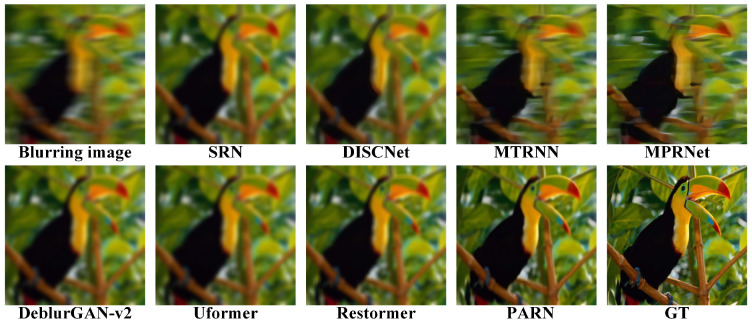
Visual comparisons on the datatset Set5 [63]. PARN restores the shaper image.

**Figure 13:**
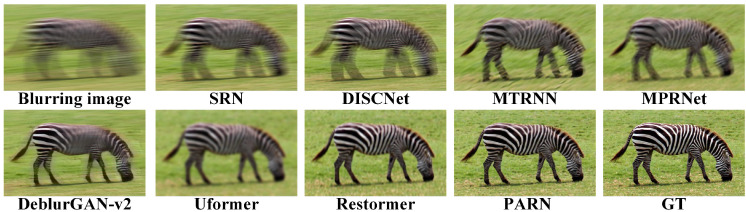
Visual comparisons on the datatset Set14 [64]. PARN better captures the context features to deblur image.

**Figure 14:**
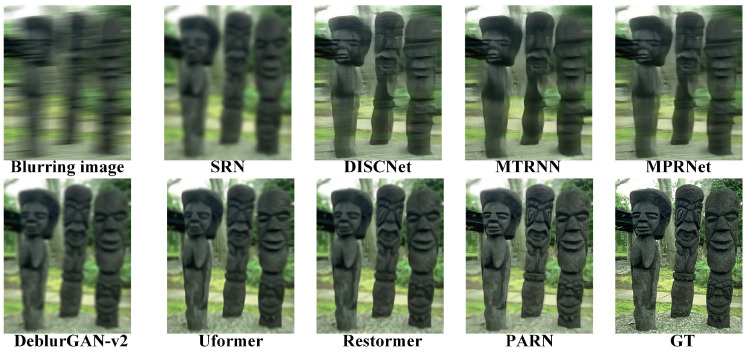
Visual comparisons on the datatset BSD100 [62]. The image restored by PARN visually closer to the GT (ground truth).

As shown in Figure 11, Figure 12, Figure 13 and Figure 14, the visual comparisons among different methods show the image is restored more shaper and more detailed by PARN. In contrast, the other methods either overly restore images and create artifacts, or not correctly remove the blur.

Overall, those metrics and visual comparisons demonstrate that the proposed method, PARN, outperforms on the synthesized datasets than other methods. The effectivity is related to the strong representations for contextualized features and guidance of the prior information for degradation.

### 3.2. Measurement Accuracy Verification

**Figure 15:**
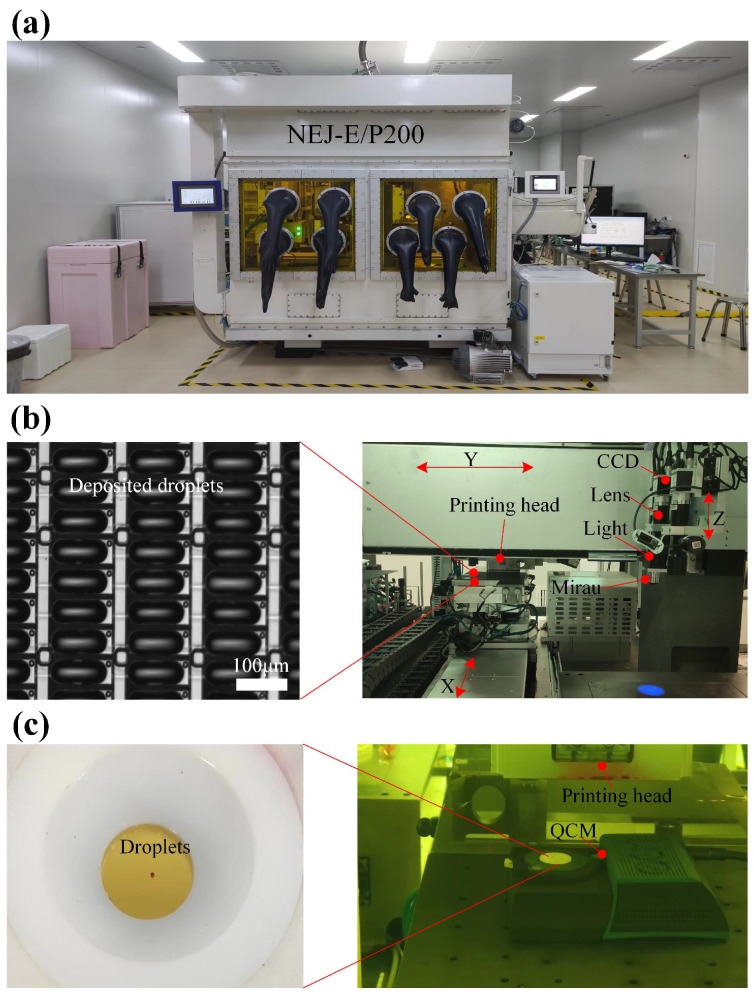
OLED inkjet printing manufacturing equipment. (**a**) Inkjet printer. (**b**) Droplet measurement system with Mirau objective and deposited droplets fabrication system. (**c**) Droplet weighing experimental setup via QCM.

In this section, the droplet volume measurement experiments are conducted. The actual working condition is shown in Figure 15a, which is OLED manufacturing equipment NEJ-E/P200 self-developed by HuaZhong University of Science Technology (HUST) and Wuhan National Innovation Technology Optoelectronics Equipment Company (NITE). With the protection of glovebox, the ink droplets are steady inkjeted on the panel, where the printing head is equiped with the YZ DOFs (degree of freedom) and the panel is equiped with the X DOF as shown in Figure 15b. Therefore, the desired printing pattern can be printed and dot matrixs can be fabricated. Besides, the measurement system is based on white light interference with a central wavelength of 600 nm. The light is split into two coherent beam by the Mirau objective, where one beam reflected from the sample is combined with the other beam to generate the interferograms recorded by CCD, and the resolution of CCD is 1224×1024 pixels and 0.28 μ m/pixel. The measurement system is equiped with YZ DOFs to capture the needed interferogram with fringes. As shown in Figure 15c, the gravimetric measurement method, quartz crystal microbalance (QCM) [68] with resolution of 0.125ng, is employed to measure the volume of droplet as the reference volume value. One hundreds small drops from one printed nozzle are sprayed onto the QCM, and the change of mass is calculated. The average volume of one single droplet is 2.275 pL. To verify the effectiveness of our measurement method, different volume of dot matrix is fabricated. In this paper, different number of drops, from one to ten, is printed by the same printed nozzle. After the dot matrix is fabricated, the measurement system captures the interferogram with fringe context.

**Figure 16:**
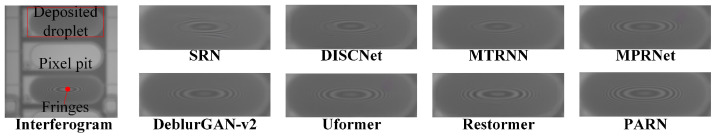
Comparison of interferogram restored by PARN and other methods.

**Figure 17:**
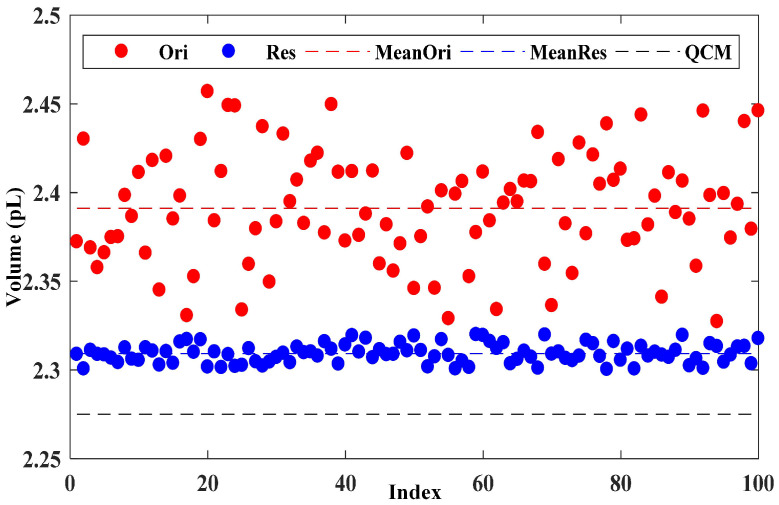
Droplet measurement results with and without PARN compared to the weighing result by QCM. Ori: without restoration. Res: restored by PARN. MeanOri: the average volume of the original group. MeanRes: the average volume of the restored group.

The visual comparisons among different methods are shown in Figure 16. The similar results are produced by retrained model, which deeper demonstrates the effectiveness of PARN. PARN restores more detail contextualized features, which is the key for CAT to caculate the volume. The measurement results are shown in Figure 17. In this paper, ten dot matrixs of droplet are fabricated by one identical choesn printing noozle. Each dot matrix contains ten deposited droplets mixed by the same number of small droplets, where the number of small droplet is from 1 to 10 corresponding to the index of group. With the aid of motor motion, CSI scans all the fabricated deposited droplets vertically and recorded all the interferograms. One interferogram for one deposited droplet is used to calculate the average volume. In Figure 17, the value of Ori means that interferogram is calculated directly by the CRN module, and CRN module maps the fringe texture in interferogram to the final volume. The value of Res means interferogram is restored by PARN before calculated by CRN module. All calculated value are compared to the refence value weighted by QCM. Therefore, there are 10 dot matrixs and 100 deposited droplets needed to be calculated. In order to verify the measurement effectiveness, the average value for each deposited droplet is shown. The reference value is 2.275 pL weighted by QCM. The value calculated with the raw image and restored image are separately shown as the Ori and the Res. The average volume of single droplet calculated with the original image is 2.396 pL. The average volume calculated after the restoration is 2.309 pL. According to the reference value 2.275 pL, the measurement errors are separately 5.3% and 1.5%. Besides, it’s obvious that the standard error of restored group is less than that of the original group. Both the standard and measurement error show that the proposed method can effectively eliminate the aberrations and restore the blur.

**Figure 18:**
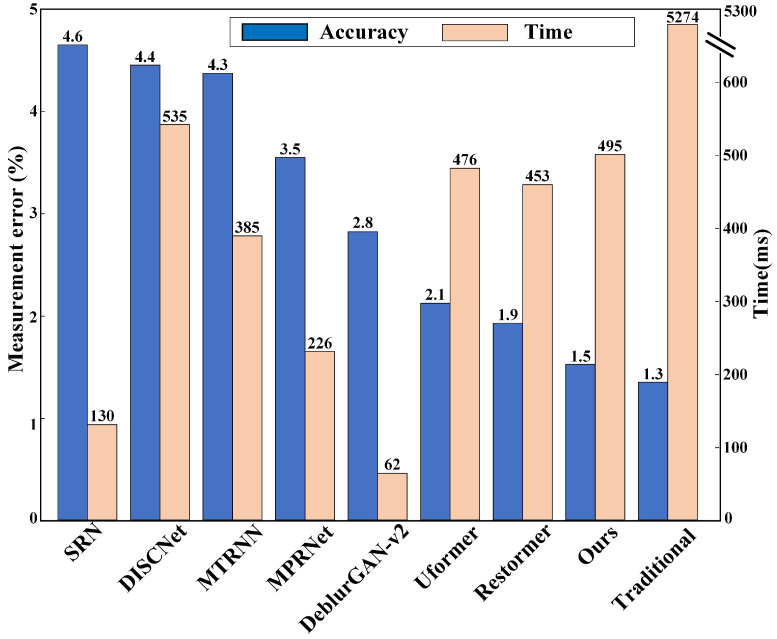
The comparisons of measurement error and time consuming for different methods and traditional scanning method.

The measurement error and time consuming are listed in Figure 18. The identical interferogram is restored by different methods, SRN, DISCNet, MTRNN, etc. All restored interferograms are input into CRN module to calculate the average volume compared to the reference volume. Firstly, the propoesd method can achieve the best accuracy among the deep learning methods. Due to the relatively simple and customized design, SRN and DISCNet has relatively low accuracy. Due to the long term contextualized representation, MTRNN gains little accuracy improvement. MPRNet based on multi-stages feature fusion restores interferogram better in fringe distribution, and achieves 3.5% measurement error. With the aid of prior information for degradation, DeblurGAN-v2 outperforms the CNN and RNN based methods. Transformer has strong representation ability for contextualized features, which is the key factor to learn the blurring conduct in global view. Therefore, Transformer based methods achieve the higher accuracy and has the best restoration effects. Due to the Zernike polynomials to discribe the aberrations, PARN can utilize the prior information of more complicated PSFs to yield 1.5% measurement accuracy. Traditional method measures the volume by the 3D morphology rebuilding. The rebuilding process demands the redundancy sampling. The measurement system scans the deposited droplet in vertical direction. During the scanning process, the interferograms are uniformly captured at different height. In this paper, the scanning distance is set to 12μ m, and the scanning speed is set to 3μ m/s. Therefore, the time for interferograms recording is about 4 s. Despite of the parameters setting related to the CCD maximum frame rate, the data acquisition time tends to a few seconds. Combined with the rebuilding time, the whole time consumption is about 10 times to the proposed method while the accuracy is similar. The traditional rebuilding methods is inefficient in data acquisition, where hundreds image will be required as shown in Figure 3 and limit the process speed. In this paper, the fringe distribution in image plane is considered, which is related to the volume of deposited droplets. With single interferogram full with fringe texture feature, the proposed method directly maps the image to the volume. Therefore, the proposed method balance the accuracy and efficiency. Besides, Owing to the inkjet printing process demands, our method can measure several to hundreds picoliter, approximately be 2-120pL, which is decided by the inkjet printing process and the pixel type.

In summary, the above experiments demonstrate the effectiveness of the proposed degradation framework and PARN in the real-world scenario of droplet measurement. Based on the complicated modeling ability of Zernike polynomials to discribe aberrations, the more real PSD can be employed. Combined with 3D imaging degradation model and ZTP module, the proposed method can utilize the prior information of degradation. With the aid of Transformer module’s strong representation ability for the context, the proposed method can restore the interferograms better. Therefore, the proposed methods succeed in achieving high droplet measurement accuracy and efficiency.

## 4. Conclusions

In order to improve the measurement efficiency of deposited droplet in CSI, an end-to-end deposited droplet volume measurement network is completed by utilizing the Newton-like interference fringe texture features in a single frame interferogram. According to the imaging characteristics of CSI, the 3D spatial imaging degradation model of interferogram is constructed, and the spatial PSF function representation is established at different scanning heights. The Zernike polynomial is used to describe different aberrations, and the ZTP mapping network is established to realize the direct mapping between Zernike space coefficient and PSF bias coefficient. To address the defocus aberrations caused by the optical system and the aberrations such as astigmatism caused by the sample, PARN is proposed based on Transformer’s excellent modeling ability, the efficiency of channel-dimension-based calculation and the multi-scale feature extraction characteristics of CNN. Through CRN encoding and decoding structure, the general features of interference fringes of the same deposited droplets at different sampling heights are extracted. Based on the extracted features, the end to end measurement of the deposited droplet volume via a single frame interferogram is completed. Without the hundreds of image recording process, VMFD calculates the volume of deposited droplet via fringe distribution directly. The time consuming is about 0.5 s, which is great progress compared to the regular CSI measurement techniques. Along with the ability to eliminate aberrations, the proposed method reaches the 1.5% accuracy. Therefore, VMFD can achieve a good balance between the accuracy and efficiency via the aid of fringe texture information.

## Figures and Tables

**Table 1:** Comparisons results on synthesized DIV2k [61], Set5 [63], Set14 [64] and BSD100 [62]. The best results are in bold font and second best results are underlined. The symbol ↑ represents the bigger value is better.

Models	DIV2K		Set5		Set14		BSD100	
	**PSNR**↑	**SSIM**↑	**PSNR**↑	**SSIM**↑	**PSNR**↑	**SSIM**↑	**PSNR**↑	**SSIM**↑
SRN [65]	22.34	0.539	23.56	0.602	22.28	0.508	21.95	0.482
DISCNet [27]	23.02	0.523	23.14	0.584	22.89	0.492	22.13	0.466
MTRNN [66]	23.65	0.542	23.78	0.605	23.51	0.51	23.32	0.493
MPRNet [34]	24.34	0.592	24.52	0.642	23.78	0.562	23.33	0.502
DeblurGAN-v2 [67]	24.15	0.584	24.35	0.635	23.56	0.558	23.27	0.496
Uformer [48]	24.84	0.625	25.21	0.672	24.78	0.606	24.25	0.542
Restormer [28]	25.04	0.668	25.85	0.723	24.80	0.602	24.68	**0.605**
**PARN**	**25.34**	**0.682**	**26.06**	**0.754**	**25.08**	**0.678**	**24.85**	0.595

## Data Availability

All data were analysed in this study. No new data were utilized.
